# Au Nanoparticles (NPs) Decorated Co Doped ZnO Semiconductor (Co_400_-ZnO/Au) Nanocomposites for Novel SERS Substrates

**DOI:** 10.3390/bios12121148

**Published:** 2022-12-08

**Authors:** Yan Zhai, Xiaoyu Zhao, Zhiyuan Ma, Xiaoyu Guo, Ying Wen, Haifeng Yang

**Affiliations:** The Education Ministry Key Lab of Resource Chemistry and Shanghai Frontiers Science Center of Biomimetic Catalysis, Shanghai Normal University, Shanghai 200234, China

**Keywords:** Co-ZnO/Au, synergistic quantum effect, Raman enhancement, tyramine, Rhodamine 6G

## Abstract

Au nanoparticles were decorated on the surface of Co-doped ZnO with a certain ratio of Co^2+^/Co^3+^ to obtain a novel semiconductor-metal composite. The optimal substrate, designated as Co_400_-ZnO/Au, is beneficial to the promotion of separation efficiency of electron and hole in a semiconductor excited under visible laser exposure, which the enhances localized surface plasmon resonance (LSPR) of the Au nanoparticles. As an interesting finding, during Co doping, quantum dots of ZnO are generated, which strengthen the strong semiconductor metal interaction (SSSMI) effect. Eventually, the synergistic effect effectively advances the surface enhancement Raman scattering (SERS) performance of Co_400_-ZnO/Au composite. The enhancement mechanism is addressed in-depth by morphologic characterization, UV-visible, X-ray diffraction, photoluminescence, X-ray photoelectron spectroscopy, density functional theory, and finite difference time domain (FDTD) simulations. By using Co_400_-ZnO/Au, SERS detection of Rhodamine 6G presents a limit of detection (LOD) of 1 × 10^−9^ M. As a real application, the Co_400_-ZnO/Au-based SERS method is utilized to inspect tyramine in beer and the detectable concentration of 1 × 10^−8^ M is achieved. In this work, the doping strategy is expected to realize a quantum effect, triggering a SSSMI effect for developing promising SERS substrates in future.

## 1. Introduction

The surface-enhanced Raman scattering (SERS) technique has superior sensitivity and affords the molecular fingerprint information of a target sample adsorbed or approaching on the surfaces of noble nanostructures (Ag, Au, and Cu), which has been widely explored in the fields of biological, pharmaceutical, contaminant, and toxin detections [1,2]. Two acceptable dominant enhancement mechanisms are the charge transfer (CT) process [3,4] and the localized surface plasmon resonance (LSPR) field, which is connection with incident laser lines [5,6]. In the literature, the greatest enhancement factor that has been reported is 10^14^, due to specific molecules located within the gaps of neighbor Ag nanoparticles, namely LSPR hot spots [7]. Nevertheless, metallic nanoparticles expose some shortcomings such as instability, expensive cost, and limited excitation wavelength [8].

As an alternative, more attention has been focused on the possibility of semiconductor materials as SERS substrates, owing to their chemical and mechanical stabilities, such as being less-poisonousness, having high photo-efficiency, and better resistance to the environment [9]. However, most semiconductors with nanostructures only contribute an enhancement factor for Raman scattering below 10^5^ [10,11]. For further improving the SERS feature of semiconductors, morphology optimization, element doping, and the composites with noble metals were investigated [12,13,14,15,16]. Amongst these, the metal and semiconductor composites exhibit the best merits because of the strong semiconductor metal interaction (SSMI) effect. Therefore, further systematical exploration of enhancement mechanisms for composites is important to design a promising SERS substrate for actual detection.

In this work, considering the similar ionic radius between Co^2+^ and Zn^2+^ ions, Co element with an optimized ratio of Co^2+^/Co^3+^ was doped in ZnO (designated as Co-ZnO), which achieved broad adsorption of the visible spectrum based on the Dopant effect [17,18]. Interestingly, when Co was doped into ZnO, quantum dots of ZnO were generated. After gold nanoparticles (Au NPs) were decorated on the surface of Co-ZnO (designated as Co-ZnO/Au), the composite showed strengthened a strong semiconductor and metal interaction (SSSMI) effect. Additionally, electron immigration increased in the interface of the metal and semiconductor, which resulted in remarkable enhancement of the LSPR effect over the whole composite. Density functional theory (DFT) and finite difference time domain (FDTD) simulations were conducted to understand the quantum-effect-advanced synergistic enhancement principle. As a real application case, by using optimal Co-ZnO/Au substrate, SERS detection of tyramine (Tyr), a kind of bioamines produced in the food-digestion process, was performed. It exhibited high detection sensitivities, with the limit of detection being around 1 × 10^−8^ mol/L.

## 2. Experimental Section

### 2.1. Reagents and Materials

Sodium hydroxide (NaOH), cobalt(II) acetate (C_4_H_6_CoO_4_), ammonium bicarbonate (NH_4_HCO_3_), tyramine (≥98%), and ZnNO_3_·6H_2_O were purchased from Sigma-Aldrich (St. Louis, MO, USA). Chloroauric acid (HAuCl_4_·4H_2_O) was bought from Sinopharm Chemical Reagent (Shanghai, China). Rhodamine 6G (R6G) was obtained from Adamas Reagent (Shanghai, China). All chemicals and reagents were of analytical grade. Ultrapure water (18.2 MΩ cm) was used throughout all experiments. Glassware was embathed in aqua regia and then thoroughly rinsed with ultrapure water. Canned beer (Tsingtao, Qingdao, China) was obtained from a supermarket.

### 2.2. Synthesis of ZnO/Au

First, 0.2 g zinc acetate was dispersed in 70 mL ultrapure water by ultrasonic wave for 30 min. A total of 10 mL of NaOH solution (2 mol/L) was added to the zinc acetate solution under constant agitation. The above solution was transferred to a reaction kettle and then put into an oven for the reaction (160 °C, 20 h). After natural cooling to room temperature, the sample was washed several times with ultrapure water to remove residual ions and molecules, and dried under a 70° vacuum. About 0.015 g of ZnO was dissolved in 25 mL of ultrapure water and heated to boiling under stirring. Finally, 1 mL of 10^−3^ M HAuCl_4_ solution was injected for 30 min under agitation until the solution turned purplish-red to obtain ZnO/Au.

### 2.3. Synthesis of Co-ZnO

Co-doped ZnO was synthesized as follows: following standard procedure, ZnNO_3_·6H_2_O (0.40 g) and C_4_H_6_CoO_4_ with different amounts including 0, 40, 120, 200, 280, 400, 480, and 600 mg were dissolved in 10 mL ultrapure water at room temperature. After 8 mL NaOH (0.5 mol L^−1^) was added, the suspension was stirred for 40 min, after which 2.4 g NH_4_HCO_3_ was added and stirred until it completely dissolved. The suspension was then dried at 60 °C for 10 h. The product was calcined in a corundum crucible with a cover at 500 °C for 2 h, followed by rapid cooling to room temperature to yield the Co-ZnO product. The obtained products were, respectively, marked as Co_40_-ZnO, Co_120_-ZnO, Co_200_-ZnO, Co_280_-ZnO, Co_400_-ZnO, Co_480_-ZnO, and Co_600_-ZnO.

### 2.4. Synthesis of Co_400_-ZnO/Au

A total of 0. 02 g of Co_400_-ZnO was dispersed in 25 mL ultrapure water, and heated to a boiling while constantly stirring. Then, 5 mL of 5% HAuCl_4_ solution was injected under stirring for 30 min until the solution turned brown-red to obtain Co_400_-ZnO/Au successfully. After cooling to room temperature naturally, Co_400_-ZnO/Au was washed with ultrapure water several times to remove residual ions and molecules, and dried at 70 °C under vacuum.

### 2.5. SERS Measurement

For SERS detection, the analyte solution was mixed with Co_400_-ZnO/Au nanocomposite suspension by a volume ratio of 1:2. Raman test was conducted by using 633 nm laser with power at 5 mW and a collection time of 3 s with 2 accumulations.

### 2.6. Instrumentation

UV-vis spectra were collected by a UV-vis spectrophotometer (SHIMADZU, UV-1800, Kyoto, Japan). The morphologies of SERS substrates were taken by a JEM-2100EXII transmission electron microscope (JEOL Co., Ltd., Tokyo, Japan), operating at 200 kV. The high-angle annular dark-field scanning transmission electron microscopy (HAADF-STEM) images and elemental mapping of SERS substrates were acquired on a Tecnai G2 S-Twin F20 field-emission transmission electron microscope (FEI, Hillsboro, OR, USA). X-ray photoelectron spectroscopy (XPS) (model PHI 5000, Versa Probe, NEC Corporation, Tokyo, Japan) was performed to identify the chemical composition of Co-ZnO/Au. X-ray diffraction (XRD) analysis was conducted on D/Max-2000 VPC (RIGAKU, Tokyo, Japan). Raman experiment was performed by using a confocal laser Raman system (Super LabRamII, Jobin Yvon, Longjumeau, France). HPLC-MS results were collected by a Q EXACTIVE PLUS HPLC-MS spectrometer (Thermo Scientific, Waltham, MA, USA).

### 2.7. Calculation Methods

The density of states (DOS) of ZnO and Co-doped ZnO were calculated by first-principle calculation based on density functional theory (DFT). The pseudopotentials and the starting DFT calculation were performed based on the Perdew–Burke–Ernzerhof (PBE) exchange-correlation functional. The plane-wave cutoff energy was set to 340 eV, and the Monkhorst-Pack method with a k-points mesh of 4 × 4 × 2 was used to sample the Brillouin-zone.

The electric field strengths of ZnO, Co_400_-ZnO, and Co-ZnO/Au were calculated by using a finite difference time domain (FDTD) method. The grid precision for FDTD simulation was 2 nm in the *X*, *Y*, and *Z* directions, and the time step was set at 200 fs. Periodic boundary conditions were applied in both the *X* and *Y* directions, while perfect matching layer boundary conditions in the *Z* direction were conducted. The plane-wave source propagated along the *z*-axis at incident wavelengths including 532, 633, and 785 nm on the nanoparticles.

## 3. Results and Discussion

### 3.1. Characterization of Co-ZnO

**Figure 1:**
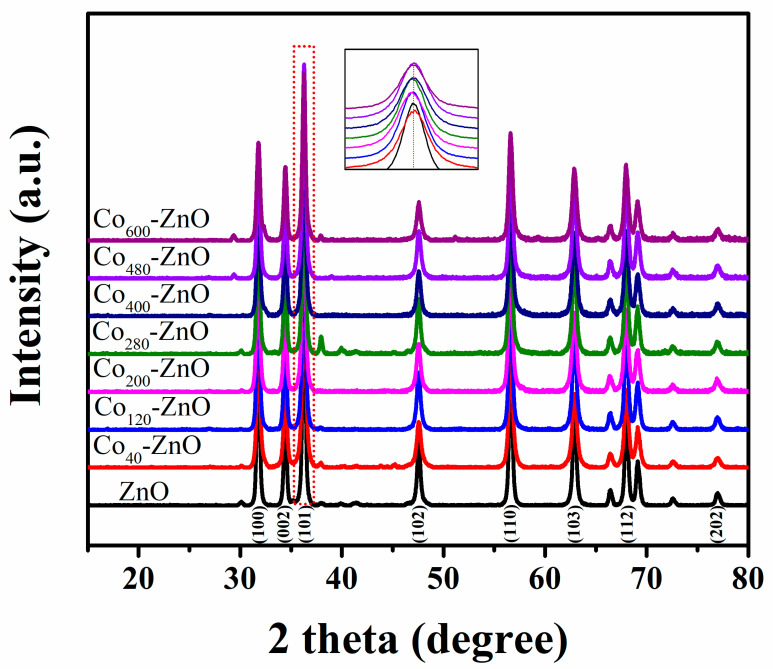
XRD patterns of ZnO and Co-ZnO.

The ionic radius of Co^2+^ (0.72 Å) is similar to that of Zn^2+^ (0.74 Å). Therefore, Co element can be easily doped into a ZnO lattice to substitute the position of Zn^2+^ ions, which avoids lattice mismatch to an extent [19,20]. In addition, the rich electronic states of Co element benefit the optimization of the magnetic, electrical, and optical properties of ZnO [21]. Consequently, the elevated impurity level caused by Co dopant shortens the energy gap of ZnO and simultaneously improves the charge-carrier separation due to creating many electron traps [22]. Herein, first, we tuned the amount of Co element in ZnO to improve SERS performance of the resultant composite. As depicted in Figure S1 of Supplementary Material, with an increasing amount of Co dopant, the color of composite ZnO materials changes from white to dark greenish. This is due to the high spin state Co^2+^ 3d_7_ (4F) involving d–d transition for oxygen coordination in tetrahedral symmetry [23,24]. In Figure 1**,** the XRD patterns of different Co-ZnO substrates display their wurtzite structures in good agreement with the JCPDS 36-1451. There being no obvious change in diffraction peaks of Co-ZnO substrates indicates that the amorphous Co oxides have a slight effect on the crystal structure of ZnO [25]. Clearly, in Figure S2 and Table S1, the crystallite size (*D*), micro strain (*ε*), and dislocation density (*ρ*) of Co doping inhibiting crystallite growth of ZnO results in a size decrease in Co-ZnO composite, which shows a connection between their differences in ionic radii and valence states [26,27]. The small size of Co-ZnO increases the surface area and boundaries, which accelerates the carrier mobility [28,29]. Additionally, elevating the amount of Co doped in ZnO initially increases the strain, resulting in alteration of the lattice constant of the composite, which is proven by the visualization of the broadened XRD peaks and slight position shifts [30,31].

XPS analysis was performed to investigate the elemental composition and chemical state. In the survey spectrum of ZnO (Figure S3), two significant peaks, centered at 1021.18 and 1044.08 eV, are attributed to the binding energies of core-level Zn 2p3/2 and Zn 2p1/2, respectively. The fitted O 1s spectrum in the ZnO matrix resolves into both peaks at 530.28 and 531.28 eV, which are, respectively, ascribed to O^2-^ ions associated with Zn^2+^ ions and O^2−^ ions in oxygen-deficient regions [32]. Obviously, in Figure S4 and Tables S2 and S3, for Co_400_-ZnO, after Co doping, the binding energy position and intensity changes in Zn^2+^ and O arise from the alternation of electron density around Zn^2+^ [33,34].

UV-vis diffuse absorption spectra provided the evidence for the substitution of Co in the ZnO lattice. In Figure S5, ZnO, when alone, showed an adsorption band at 392 nm. In the case of Co-ZnO, the red shift of the band edge (marked with the arrow in Figure S6) indicates the decrease in band gap energy [35]. Detailed information involving the band gap (Eg) was estimated by Tauc formula, [36] and the optical absorption edge (nm) of pure ZnO and Co-ZnO samples is tabulated in Table S4. Co_400_-ZnO presents the highest absorption edge at 479 nm and the broadest visible absorption region, which peaked at 567, 612, and 654 nm, corresponding to the d–d transitions of the Co ions, [18] showing that visible light excitation in the solar spectrum could generate more electron–hole pairs within Co_400_-ZnO [37,38].

In Figure S7 and Table S4, compared with ZnO, the CT process between d electrons of the Co element and the conduction band (CB) or valence band (VB) of ZnO decreases the band gap for Co-ZnO composites, and Co_400_-ZnO has the lowest band gap. The diagram of VB-XPS spectra for the band structure evolution of Co-doped ZnO samples are given in Figures S8 and S9. The ease degree of electrons jumping from the VB to the CB is closely dependent on the band gap width [39,40]. In Figure S10, the corresponding calculated density of states (DOS) is consistent with the experiment results. The VB width of Co-ZnO is slightly increased compared to ZnO alone, implying mobility enhancement of the hole. Identically, the broadened CB also suggests the accelerating electron mobility [41,42].

Photoluminescence (PL), as a direct method for estimating the recombination rate of photo generated charge pairs in the crystal structure, is related to lattice defects and surface states [43]. High intensity in the PL signal indicates a rapid recombination rate of charge carriers, resulting in poor SERS performance [44]. The PL emission spectra of the samples were recorded by using an excitation wavelength of 233 nm. In Figure S11, comparably, the lowest PL signal from Co_400_-ZnO samples can be attributed to the coexistence of Co^3+^ and Co^2+^, with the ratio of 0.9578 greatly inhibiting the recombination between electron–hole pairs.

The chemical structure of the Co-ZnO composites was also studied by the Fourier transform infrared (FTIR) method. In Figure S12, for pristine ZnO, the FTIR bands at 1438, 1649, and 3450 cm^−1^ belong to -OH deforming, O-H stretching, and -OH stretching, respectively [45]. After Co-doping, the FTIR bands regarding ZnO vibrations shift to a low wavenumber because of a partial electron transfer between ZnO and Co [46]. One of the possible principles is that defects produced in ZnO by introducing Co could act as electron traps and become an intermediate state of electron transfer bridge [47,48], which would improve photon-induced charge transfer (CT) and the photo-generated charge carrier separation efficiency.

The Raman spectra of R6G (10^−6^ M) on Co-ZnO/Au substrates in Figure S13 indicate that the resultant optimal Co_400_-ZnO could greatly improve the separation efficiency of electron and hole under visible light excitation. Furthermore, the Co_400_-ZnO/Au composite, as the SERS substrate, exhibits a long-term stability and remarkable detection sensitivity.

The photon-induced charge-transfer mechanism of Co-ZnO is shown in Figure S14A. Obviously, for ZnO alone, the visible light hardly excites the electrons from the VB to CB because of the large band gap between the highest occupied molecular orbital (HOMO) and the lowest unoccupied molecular orbital (LUMO) level of the target molecules. In the case of Co-ZnO, the narrowed band gaps would benefit electronic transitions from the VB of ZnO to the surface state energy level (Ess) [49,50], and the electrons would then be injected into the LUMO of the adsorbed molecules.

A conceivable energy level diagram with the carrier transfer mechanism is displayed in Figure S14B. The Co^2+^ ion is unstable owing to easy loss of d7 electronic configuration to Co^3+^ (d7). In detail, Co^2+^ tends to transform electrons to the surface absorbed oxygen (Equation (2)) [51] and, simultaneously, to the formation of superoxide (⋅O_2_^−^). The Co^3+^ tends to convert to Co^2+^ (Equation (3)) by capturing the photo-induced electrons. In the case of a low amount of Co dopant, the occurrence of Co^2+^ ions as electron traps enhances the separation of electron and hole. However, at a higher concentration of Co dopant, with the ratio of Co^2+^/Co^3+^ decreasing, the availability of electron traps descends due to excessive Co^3+^ ions with vacancies as novel centers, facilitating the recombination of electrons and holes.

ZnO + hv → e − CB + h+ VB (1)

Co^2+^ + O_2_ → Co^3+^ + ⋅O_2_^−^ (2)

Co^3+^ + e − CB → Co^2+^ (3)

In all, the due ratio of Co^2+^/Co^3+^ in Co_400_-ZnO composite correspondingly resulted in the smallest grain size, the narrowest band gap, the lowest PL intensities, and superior light absorption capability. As mentioned above, the CT mechanism of Co_400_-ZnO composite is the dominant contribution to the following superior Raman enhancement of target molecules. Therefore, Co_400_-ZnO was chosen to prepare Co_400_-ZnO/Au as the next SERS substrate.

### 3.2. Characterization of Co-ZnO/Au

**Figure 2:**
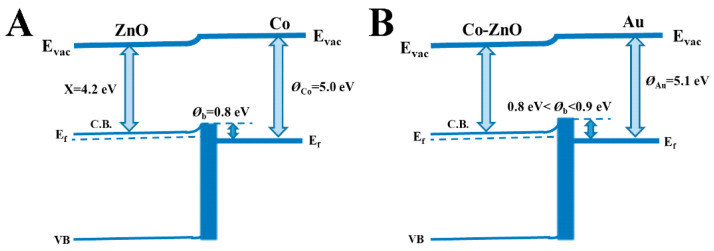
Proposed band structures of the Co_400_-ZnO (**A**) and Co_400_-ZnO/Au (**B**).

UV-vis diffuse spectra of Co_400_-ZnO and Co_400_-ZnO/Au (Figure S15) show successful preparation of Co_400_-ZnO/Au substrate due to the occurrence of a SPR band at 523 nm from Au nanoparticles. The hydrothermal preparation protocol was employed to synthesize a three-dimensional Co_400_-ZnO/Au composite. SEM and TEM images (Figure S16) reveal that the morphology of Co_400_-ZnO is cylindrical and the Co_400_-ZnO/Au is a Coral-shaped porous structure. In Figure S17, compared with Co_400_-ZnO, broadened XRD patterns for Co_400_-ZnO/Au at 31.66° and 34.22° with a slight shift indicate the partial incorporation of Au element into the crystal lattice of Co_400_-ZnO [52]. Owing to the fact that the Fermi energy of ZnO is lower than Co and Au, the modification of gold species changes the charge distribution and, then, the electron transfer on the surface, to achieve balance state [53]. As a result, a remark of numerous free electrons on the boundaries between metal and semiconductors is conducive to enlarging the localized SPR (LSPR) effect [41,54]. The detailed band structure distributions of the Co_400_-ZnO and Co_400_-ZnO/Au are illustrated in Figure 2.

**Figure 3:**
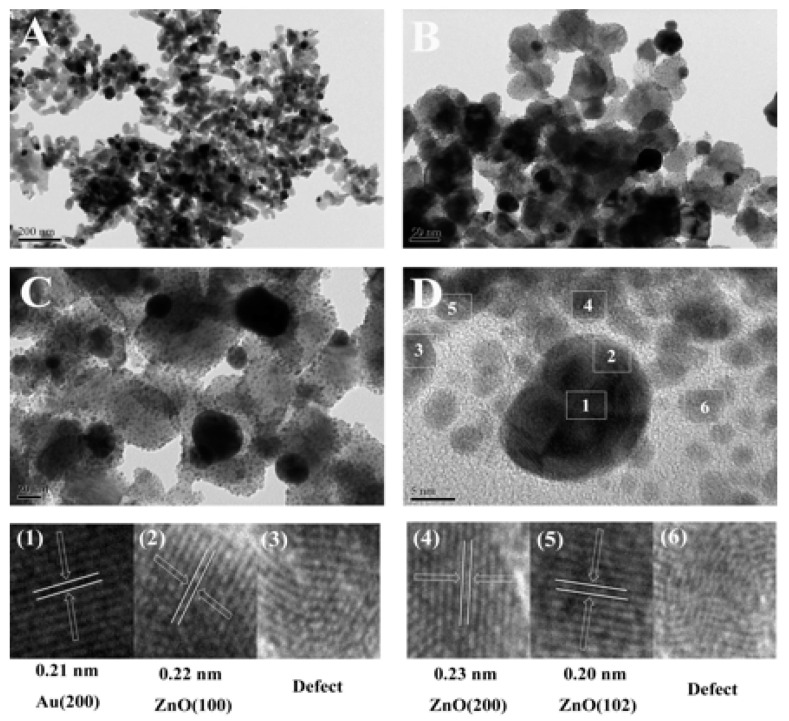
(**A**–**C**) TEM images of Co_400_-ZnO/Au at different scales. (**D**) Representative high-resolution TEM images at the interface of Co_400_-ZnO/Au (the regions indexed below the TEM image correspond to the marked areas by numbers 1–6).

In Figure 3D, the lattice spacing merits of ZnO indicate the presence of stacking faults and defects. Clearly, in Figure S18, there are a large amount of quantum dots (QDs) of ZnO, ranging from 2.3 to 3.3 nm, generated in the Co_400_-ZnO/Au composite, which should contribute to the quantum confinement effect [55]. According to the Hamiltonian of semiconductors, in the presence of ZnO QDs, very high mobility of charge carriers leads to the fusing of exciton and plasmon resonances [56].

**Figure 4:**
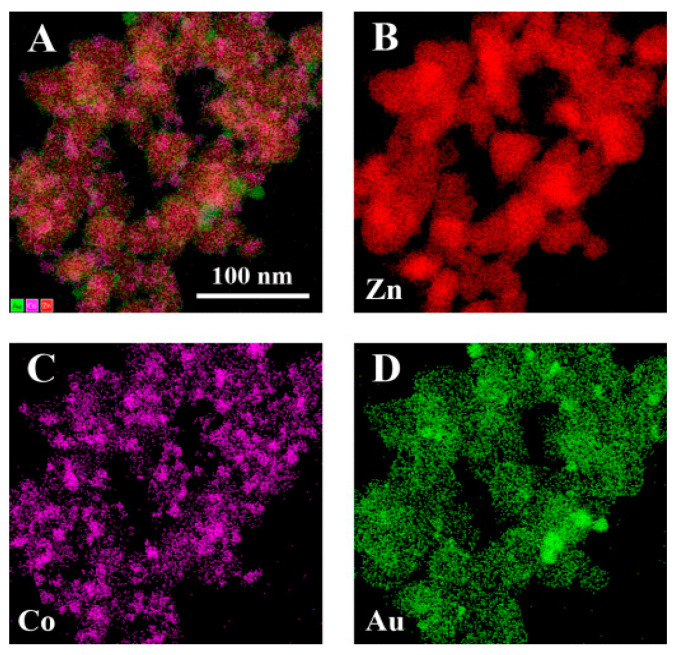
EDX elemental mappings of Co_400_-ZnO/Au: The overlaid image (**A**) from (**B**−**D**), and Zn (**B**), Co (**C**), and Au (**D**).

The corresponding energy-dispersive X-ray (EDX) elemental mapping images (Figure 4) and TEM-EDS results (Figure S19) of Co_400_-ZnO/Au were recorded to confirm the uniform distribution of the Co, Zn, and Au elements.

On the other hand, QDs with many defects and a lack of long-range atomic order [57,58] further strengthen the strong semiconductor metal interaction (SSSMI) effect within Co_400_-ZnO/Au composite. The HRTEM images demonstrate that the particles tightly contacted to form an interfacial hetero junction, efficiently retard the recombination of photo-generated electron/hole pairs, reduce the photo-generated charge diffusion length [59,60], and augment the exposure area of active sites. Therefore, quantum confinement inducing the SSSMI effect enabled Co_400_-ZnO/Au to provide a greater SERS effect.

**Figure 5:**
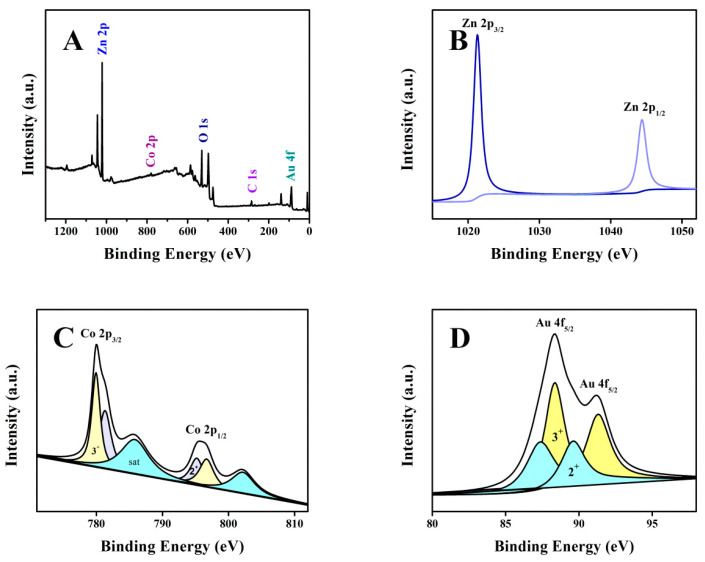
XPS spectra of (**A**) XPS survey spectrum of Co_400_-ZnO/Au, (**B**) Zn 2p, (**C**) Co 2p, and (**D**) Au 4f for Co_400_-ZnO/Au.

In the XPS results, shown in Figure 5A and in Table S5, compared with ZnO and Co_400_-ZnO, the binding energies of Zn, O, and Co in Co_400_-ZnO/Au shift, demonstrating the intra-atomic CT process [61]. In Figure 5B, for the XPS spectrum of Zn2p in Co_400_-ZnO/Au, the binding energies of Zn 2p_3/2_ and Zn 2p_1/2_ present at 1021.3 and 1044.3 eV, respectively [62]. Notably, the binding energy of Zn 2p in Co_400_-ZnO/Au showed a positive shift of 0.31 eV in comparison to 1044.08 eV of Zn 2p in Co_400_-ZnO (Figure S3), further proving the strengthened strong semiconductor metal interaction (SSSMI) effect between ZnO and Au NPs [63]. In Figure 5C, a faint Co2p central peak appears in the span from 775 to 800 eV. In detail, two binding energies of Co2p_3/2_ and Co2p_1/2_ orbitals were located at 781.2 and 796.7 eV, respectively. A jolting companion peak at 786 eV is indicated as Co^2+^ [64,65]. In Figure 5D, for Co_400_-ZnO/Au, XPS bands at 87.38 and 88.38 eV, corresponding to electronic states of Au^2+^ (minor amount) and Au^3+^ (high amount), hint at the abundant free electrons in the composite. Additionally, the binding energy of the Au4f_5/2_ in the composite centered at 88.38 eV, shifts (the standard XPS peak of Au4f_5/2_ positioned at 87.4 eV), which is also due to the SSSMI effect [66]. The electron exchange between Au^3+^ and Co^2+^ ions is given as follows:

Au^3+^ + Co^2+^ = Au^2+^ + Co^3+^ (4)

### 3.3. Simulation of Electromagnetic Field Enhancement

**Figure 6:**
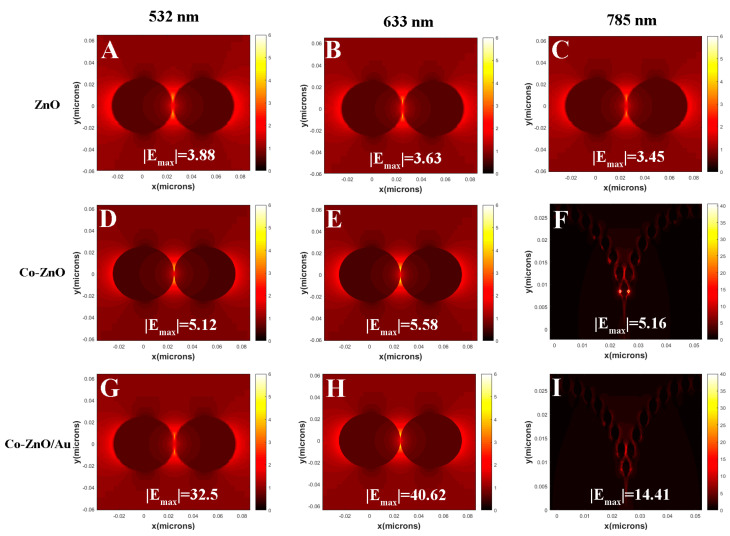
Electromagnetic field enhancement of ZnO, Co−ZnO, and Co−ZnO/Au nanostructures by using finite difference time domain simulations: (**A**–**C**) ZnO structure in xy axial under 532 nm laser; (**D**–**F**) Co−ZnO structure in xy axial under 633 nm laser; (**G**–**I**) ZnO structure in xy axial under 785 nm laser.

The FDTD simulation was used to simulate the surface electric field distribution of ZnO, Co-ZnO, and Co-ZnO/Au under exposure to lasers at 532, 633, and 785 nm. As shown in Figure 6, under irradiation with a 633 nm laser, the electric field enhancement factor of Co-ZnO can reach about six at the gap of neighboring nanoparticles, which is due to the Co doping effectively changing the photoelectric properties in comparison with the case of ZnO alone.

When it comes to Co-ZnO/Au, the SSSMI effect between ZnO QDs and AuNPs contributes to a great enhancement of the electric field, and an enhancement factor approximately equal to 40 could be reached, which is seven-fold greater than Co_400_-ZnO, shown Figure S20. Figure S20 shows the concentration-dependent SERS spectra of R6G solutions recorded on Co_400_-ZnO. Clearly, Co_400_-ZnO, due to the CT mechanism, could also contribute to the Raman signal enhancement of target molecules, to an extent.

The FDTD simulation is validated by the SERS results of 10^−7^ mol/L R6G acquired on Co_400_-ZnO/Au under different irradiations with 532, 633, and 785 nm lasers. Clearly shown in Figure S21, the matching of the 633 nm laser to the electromagnetic resonance absorption of the Co_400_-ZnO/Au substrate contributes the greatest SERS signal [67]. As shown in Figure S22A, by using Co_400_-ZnO/Au, the limit of detection (LOD, determined on the ratio of the signal to noise (S/N) equaling to 3) for R6G is 1 × 10^−9^ mol/L.

### 3.4. Co_400_-ZnO/Au-Based SERS Detection of Tyr

Tyramine (Tyr), as one of bioamines, is commonly produced in food and beverage as a consequence of microorganism fermentation and decomposition processes [68]. Overdose of Tyr from food stuffs taken by a person results in various adverse physiological effects such as hypertension, rash, cardiac palpitation, intracerebral hemorrhage, and even death in some severe cases [69]. The European Union poses a maximum limitation of Tyr content of 100–800 mg/kg in foods. Routinely, liquid chromatographic-fluorescence detectors (LC-FLD) [70] and liquid chromatographic-mass spectrometry (LC-MS) [71] are employed to analyze Try residue in foods. However, LC-based methods suffer from tedious sample pre-concentration, reagent-consumption, and the need for well- training persons.

**Figure 7:**
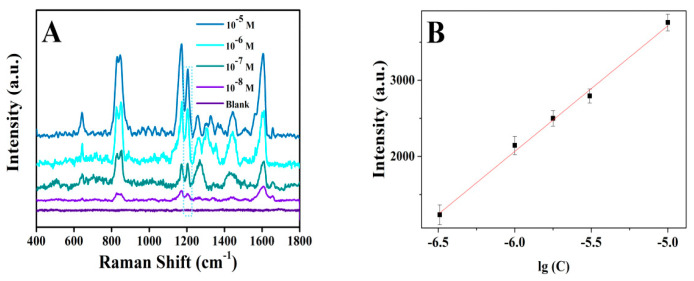
(**A**) Concentration-dependent SERS spectra of tyramine recorded on Co_400_-ZnO/Au substrate. (**B**) Calibration plot based on Raman intensity at 1208 cm^−1^.

As shown in Figure S22B, Co_400_-ZnO/Au has the strongest Raman enhancement effect for Try. Concentration-dependent SERS spectra of Tyr, using Co_400_-ZnO/Au, are shown in Figure 7A and the normal Raman spectrum of powder Tyr is also given in Figure S23. Figure 7B shows a linearity concentration relationship ranging from 1.0 × 10^−8^ to 1 × 10^−5^ mol/L, with the correlation coefficient of 0.9838 based on the characteristic band intensity at 1208 cm^−1^. Tyr, with a concentration at 1 × 10^−8^ M, could be detectable, which meets the detection sensitivity requirement of the EU for total tyrosine content in foods. In Figure S24, the relative standard derivation (RSD) of the SERS intensities at 613 cm^−1^ recorded from 20 randomly selected points on Co_400_-ZnO/Au substrate is 8.05%, which indicates a reasonable signal uniformity. After storage in ambient conditions for 70 days, the Raman signal recorded on Co_400_-ZnO/Au substrate kept 90% of its level of signal intensity obtained on freshly prepared substrate, exhibiting excellent shelf-time (Figure S25). We can obtain reproducible SERS spectra of R6G (10^−6^ M) on the three batches prepared Co_400_-ZnO/Au substrates in Figure S26.

**Figure 8:**
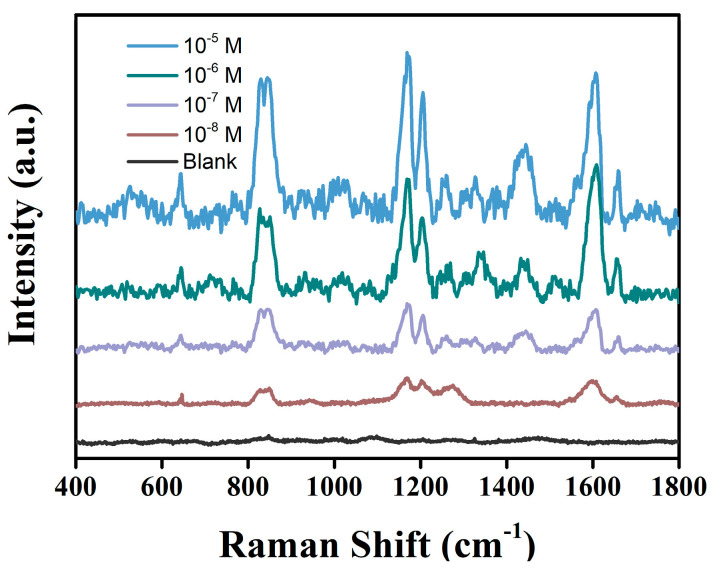
Concentration-dependent SERS spectra of tyramine in beer on Co_400_−ZnO/Au substrate.

As shown in Figure 8, in beer, tyramine at concentration as low as 1 × 10^−8^ M can be detected. As shown in Table 1, the relative standard deviation is 0.29~5.05%, and the reasonable recovery is 91.20~107.15%. In Table 2, compared with the other assays for Tyr in the literature, the Co_400_-ZnO/Au-based SERS method shows a good sensitivity.

## 4. Conclusions

In summary, the resultant optimal Co_400_-ZnO could reasonably improve the separation efficiency of electron and hole under visible light excitation. Furthermore, the Co_400_-ZnO/Au composite was prepared as an SERS substrate, which exhibited a long-term stability and a remarkable detection sensitivity for R6G with the LOD being as low as 1 × 10^−9^ M. Based on XPS characterization, DFT simulation, and FDTD theoretical exploration, this promising SERS effect can be attributed to the doping of Co to generate ZnO semiconductor with many defects accompanying the formation of certain QDs, triggering SSSMI between ZnO QDs and AuNPs. The synergistic effect boosted the huge localized electromagnetic field. As a real application case, by using Co_400_-ZnO/Au-based SERS assay, the lowest detectable concentration was 1 × 10^−8^ M. In this work, an effort was made to explore whether the composite of noble metal and semiconductor quantum dots could be developed as the excellent SERS substrate for trace detection.

## Figures and Tables

**Table 1:** Detection recovery of tyramine in Beer by Co_400_-ZnO/Au-based SERS.

Samples	ADD (umol/L)	SERS (M) (umol/L)	Recovery (%) ± SD
1	10	10.23	102.33 ± 1.03
2	1	0.912	91.20 ± 5.05
3	0.316	0.338	107.15 ± 1.84
4	0.1	0.095	95.50 ± 0.29

**Table 2:** Comparison with other methods for the determination of Tyramine.

Method	Substrates	Linear Range (moL/L)	LOD (moL/L)	Real Sample	Reference
Raman	Co_400_-ZnO/Au	10^−5^–10^−8^	1 × 10^−8^	Beer	This work
Molecularly Imprinted	Fe_3_O_4_@SiO_2_-MPS@MIP	5.4 × 10^−4^–1 × 10^−6^	1.8 × 10^−7^	Beer	[72]
Electrochemistry	Ag-substituted ZnO modified GCE	9 × 10^−4^–1 × 10^−6^	2.72 × 10^−7^	Beer	[73]
Electrochemistry	poly-TB modified carbon SPE	2.7 × 10^−4^–2 × 10^−8^	2 × 10^−8^	-	[74]
Electrochemistry	poly(His)/SPGE	2 × 10^−5^–5 × 10^−7^	2.2 × 10^−7^	Cheese	[75]

## Data Availability

The data that support the findings of this study are available from the corresponding author upon reasonable request.

## Supplementary Materials

The following supporting information can be downloaded at: https://www.mdpi.com/article/10.3390/bios12121148/s1. Figure S1: The digital pictures of the as-prepared different percent of Co-doped ZnO samples; Figure S2: (a) Crystallite size and (b) micro strain and dislocation density for all the samples investigated in this study (from 1 to 8: ZnO, Co_40_-ZnO, Co_120_-ZnO, Co_200_-ZnO, Co_280_-ZnO, Co_400_-ZnO, Co_480_-ZnO, Co_600_-ZnO, respectively); Figure S3: (A) XPS survey spectrum of ZnO, (B) and (C) XPS spectra of Zn 2p and O for ZnO; Figure S4: XPS spectra of (A) XPS survey spectrum of Co_400_-ZnO, (B) Zn 2p (C) Co 2p, and (D) O 1s for Co_400_-ZnO; Figure S5: UV-vis diffusion reflectance spectra of Au(A) and ZnO/Au(B) samples; Figure S6: UV-vis diffusion reflectance spectra of ZnO and Co-ZnO samples; Figure S7: Band gaps for the as-prepared different percent of Co-doped ZnO samples; Figure S8: VB-XPS spectra of Co-doped ZnO samples; Figure S9: Schematic band structure evolution of Co-doped ZnO samples; Figure S10: The density state of (A) pristine ZnO and (B) Co substitution in ZnO lattice. The dotted lines at energy zero represent the Fermi level; Figure S11: PL spectra at the excitation wavelength of 233 nm of ZnO and Co- doped ZnO samples; Figure S12: FTIR spectra of ZnO and Co-doped ZnO samples; Figure S13: The Raman spectra of R6G(10^−6^ M) on Co-ZnO/Au substrates; Figure S14: Band structure of the ZnO (A) and change in the band structure of ZnO by Co dopant; Figure S15: UV-vis diffusion reflectance spectra of Co_400_-ZnO and Co_400_-ZnO/Au samples; Figure S16: (A,B) TEM images of Co_400_-ZnO. (C) SEM image of Co_400_-ZnO. (D) SEM image of Co-ZnO/Au; Figure S17: XRD patterns of ZnO, Co_400_-ZnO, and Co_400_-ZnO/Au. (A) Wide-angle patterns and (B) Selected-angle patterns; Figure S18: Size distribution of ZnO quantum dots in Co_400_-ZnO/Au; Figure S19: TEM-EDS result of Co-ZnO/Au; Figure S20: Concentration-dependent SERS spectra of R6G solutions recorded on Co_400_-ZnO; Figure S21: SERS spectra of R6G (10^−7^) recorded on Co_400_-ZnO/Au under different irradiations with 532, 633, and 785 nm lasers; Figure S22: (A) SERS spectra of R6G molecules adsorbed onto Co-ZnO/Au. (B) SERS spectra of Tyr (1 × 10^−8^ M) on Co-ZnO/Au, ZnO/Au, and Au NPs; Figure S23: Normal Raman spectrum of powder Tyramine; Figure S24: (A) SERS spectra of R6G (10^−6^ M) recorded from 20 randomly- selected points on Co_400_-ZnO/Au. (B) The statistic on Raman Intensities of R6 G (10^−6^ M) recorded from 20 randomly- selected points on Co_400_-ZnO/Au; Figure S25: Raman intensities of R6G (10^−6^ M) by using Co_400_-ZnO/Au monitored during storage in ambient condition for 90 days; Figure S26: The SERS spectra of R6G (10^−6^ M) recorded on the three batches of Co_400_-ZnO/Au substrate; Table S1: Summary of Crystallite size (D), Dislocation density (*ρ*), and Micro strain (ε); Table S2:. Binding energy (BE) of Co in Co-ZnO samples; Table S3: Binding energy (BE) of Zn and O in Cox-ZnO samples; Table S4: The band gap (Eg) and optical absorption edge (nm) of pure ZnO and Co-ZnO samples; Table S5: Binding energy (BE) of ZnO, Co_400_-ZnO and Co_400_-ZnO/Au.
