# Supplementary material for: Au Nanoparticles (NPs) Decorated Co Doped ZnO Semiconductor (Co400-ZnO/Au) Nanocomposites for Novel SERS Substrates
Source: Biosensors (Basel). 2022 Dec 8;12(12):1148. doi: 10.3390/bios12121148 (PMC9775326; doi:10.3390/bios12121148)
Supplement: Supplementary file 1 [file biosensors-12-01148-s001.zip › biosensors-2035793-supplementary.pdf]

Supplementary Information

Au nanoparticles (NPs) decorated Co-doped ZnO semiconductor (Co400-ZnO/Au) nanocomposites for novel SERS substrates

Yan Zhai [#], Xiaoyu Zhao[#], Zhiyuan Ma, Xiaoyu Guo, * Ying Wen and Haifeng Yang*

The Education Ministry Key Lab of Resource Chemistry and Shanghai Frontiers Science Center of Biomimetic Catalysis, Shanghai Normal University, Shanghai 200234, P. R. China.
*Correspondence authors: E-mail: hfyang@shnu.edu.cn (Haifeng Yang); gxy2012@shnu.edu.cn (Xiaoyu Guo).

#Equal contribution in this research

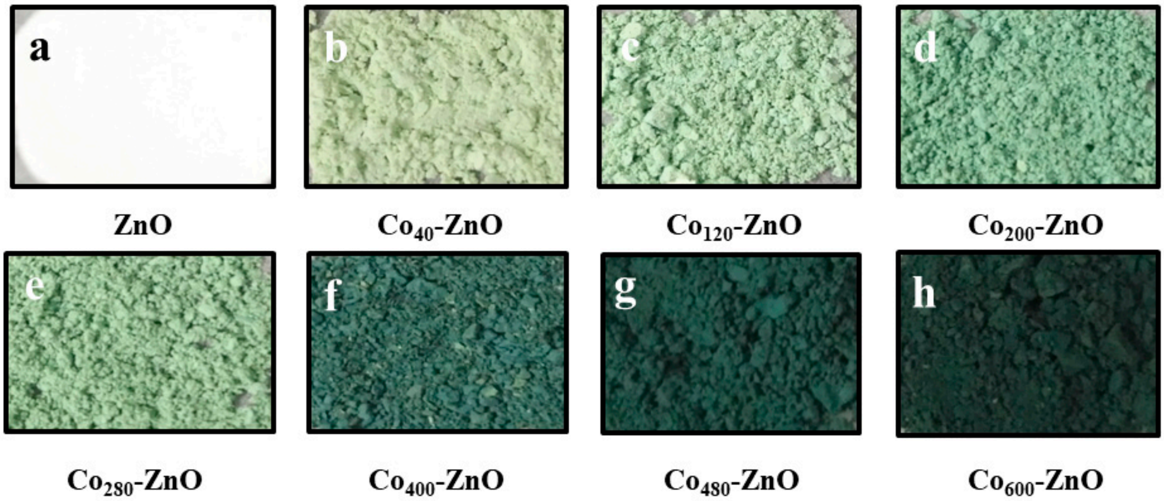

Figure S1. The digital pictures of the as-prepared different percent of Co-doped ZnO samples.

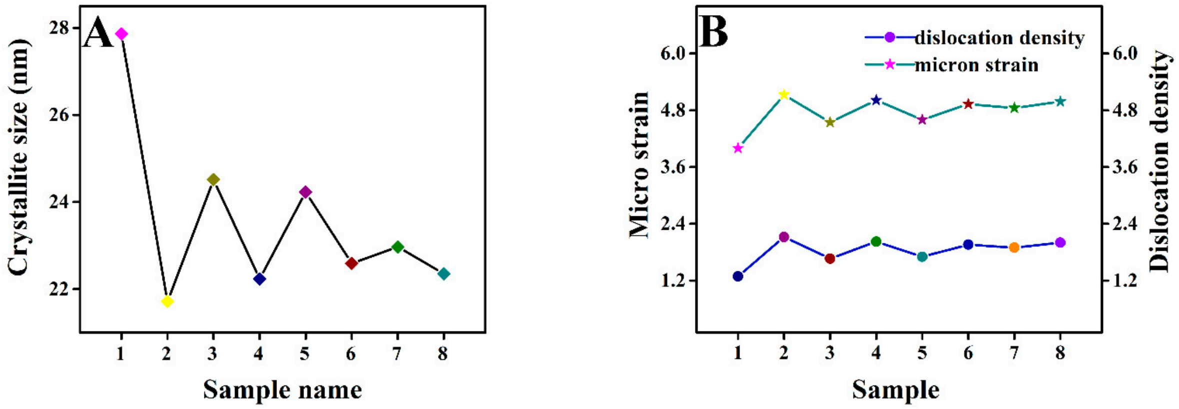

Figure S2. (a) Crystallite size and (b) micro strain and dislocation density for all the samples investigated in this study (from 1 to 8 ： ZnO, Co_{40}-ZnO, Co_{120}-ZnO, Co_{200}-ZnO, Co_{280}-ZnO, Co_{400}-ZnO, Co_{480}-ZnO, Co_{600}-ZnO, respectively).

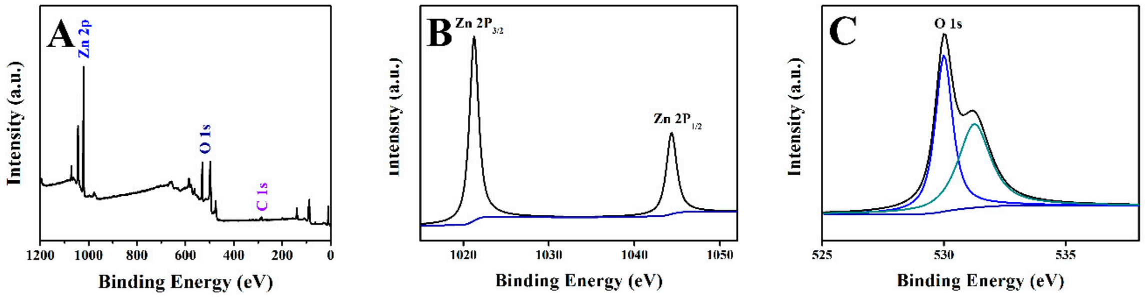

Figure S3. (A) XPS survey spectrum of ZnO, (B) and (C) XPS spectra of Zn 2p and O for ZnO.

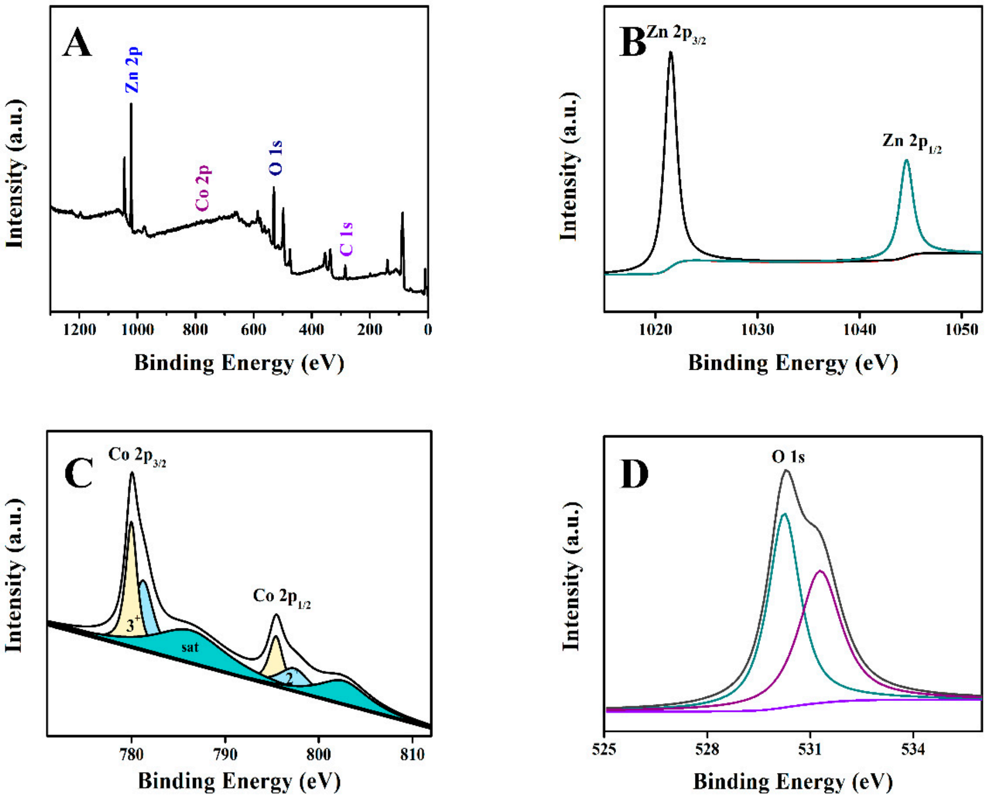

Figure S4. XPS spectra of (A) XPS survey spectrum of Co_{400}-ZnO, (B) Zn 2p (C) Co 2p, and (D) O 1s for Co_{400}-ZnO.

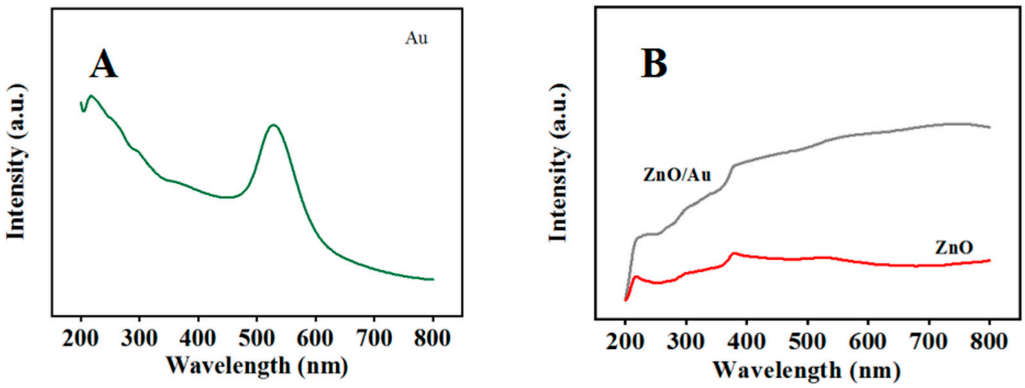

Figure S5. UV-vis diffusion reflectance spectra of Au(A) and ZnO/Au(B) samples.

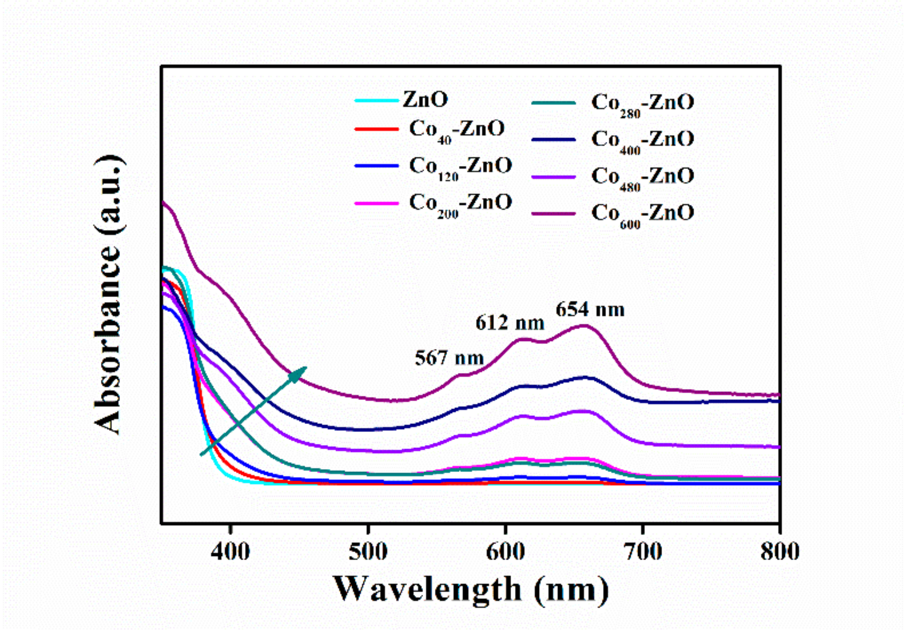

Figure S6. UV-vis diffusion reflectance spectra of ZnO and Co-ZnO samples.

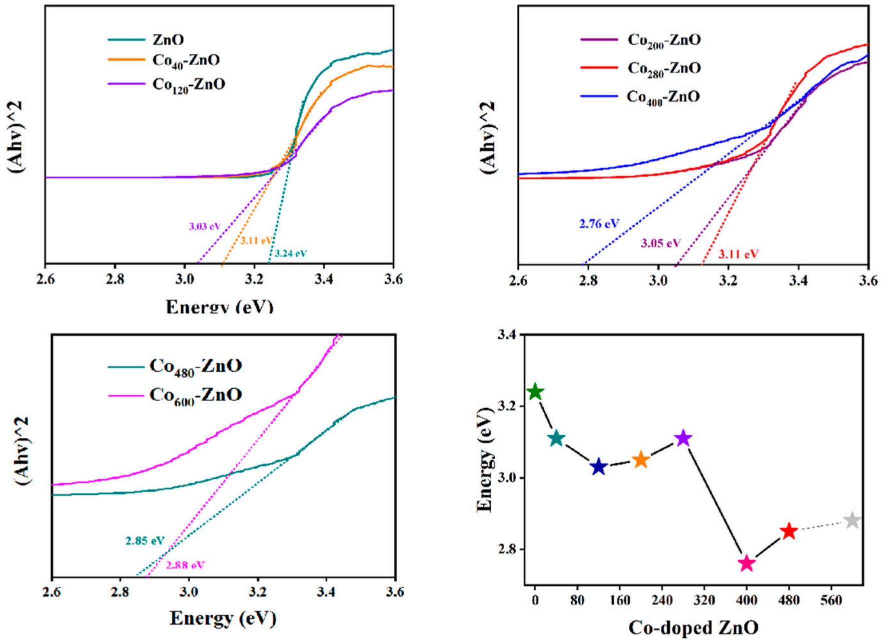

Figure S7. Band gaps for the as-prepared different percent of Co-doped ZnO samples.

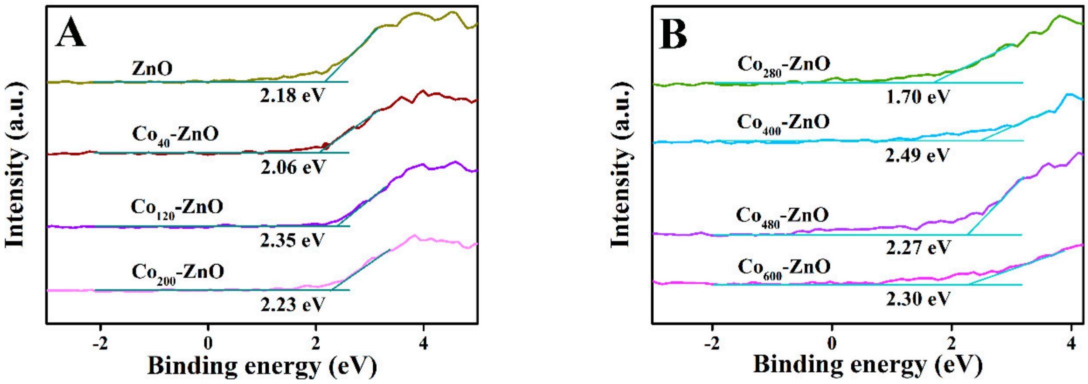

Figure S8. VB-XPS spectra of Co-doped ZnO samples.

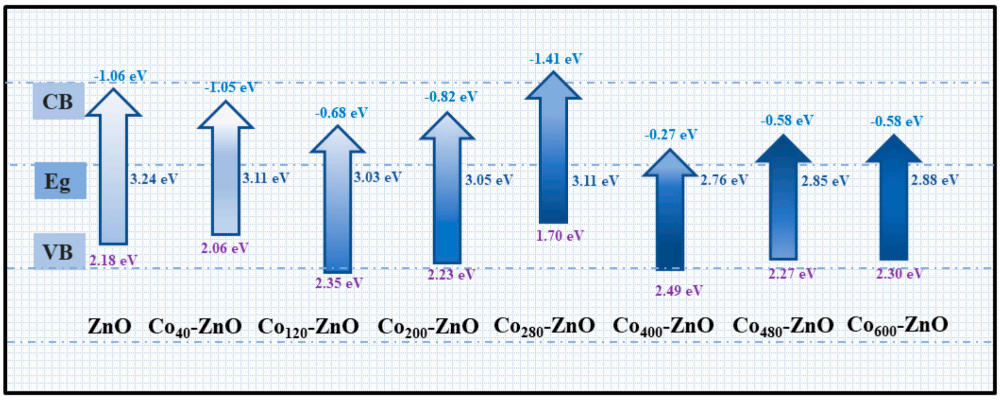

Figure S9. Schematic band structure evolution of Co-doped ZnO samples.

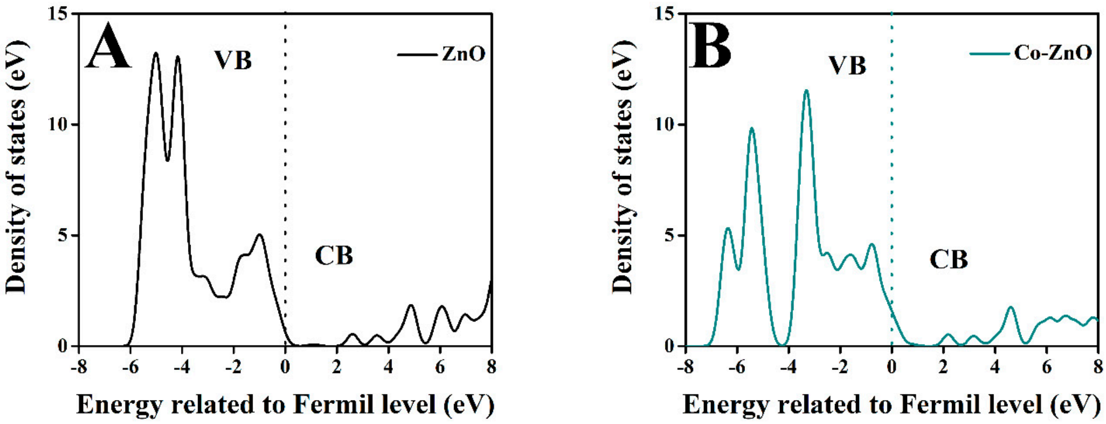

Figure S10. The density state of (A) pristine ZnO and (B) Co substitution in ZnO lattice. The dotted lines at energy zero represent the Fermi level.

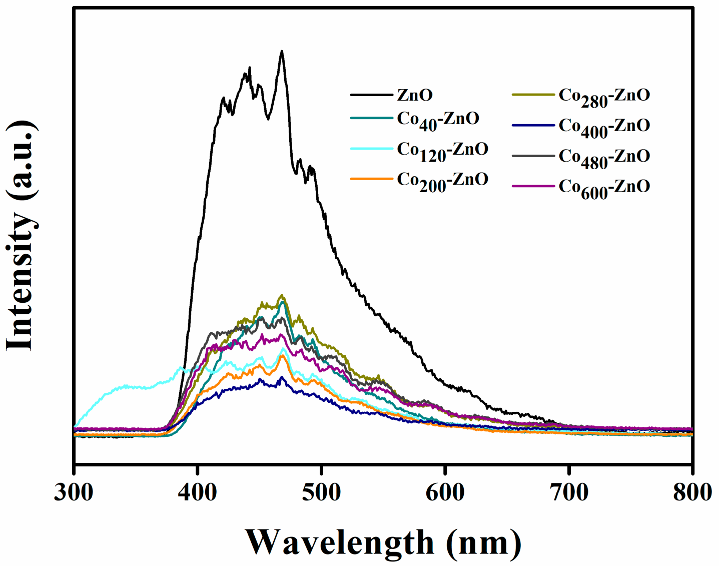

Figure S11. PL spectra at the excitation wavelength of 233 nm of ZnO and Co-doped ZnO samples.

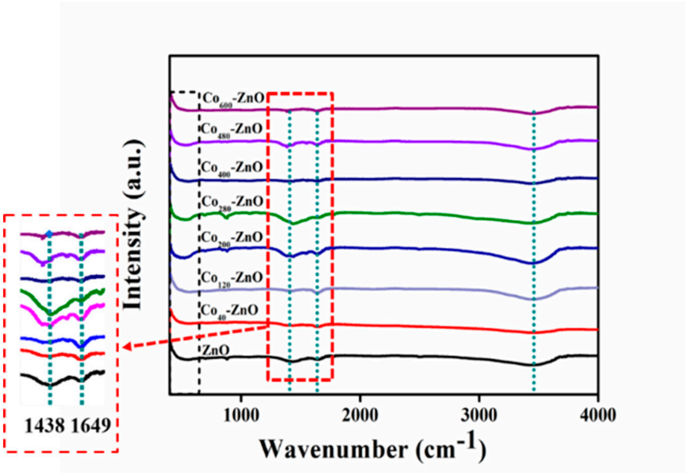

Figure S12. FTIR spectra of ZnO and Co-doped ZnO samples.

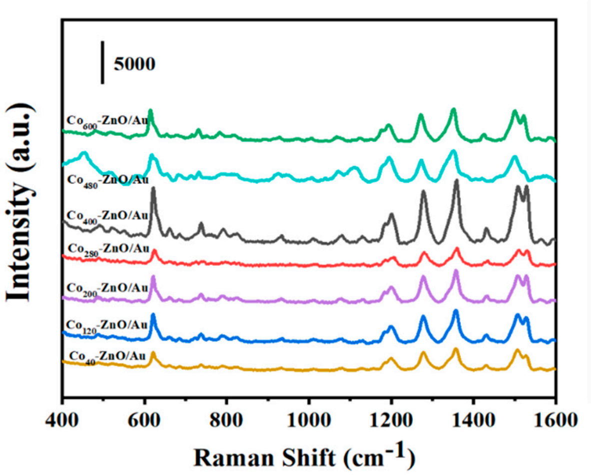

Figure S13. The Raman spectra of R6G(10^{-6} M) on Co-ZnO/Au substrates.

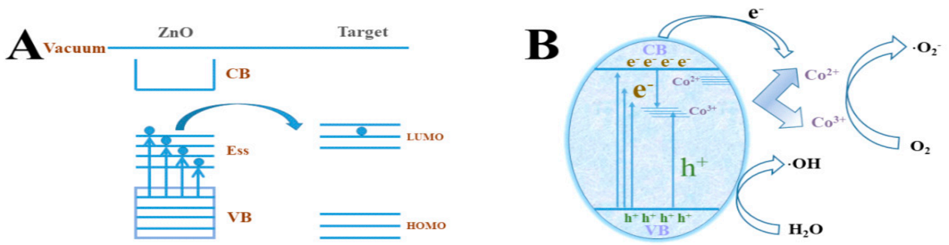

Figure S14. Band structure of the ZnO (A) and change in the band structure of ZnO by Co dopant.

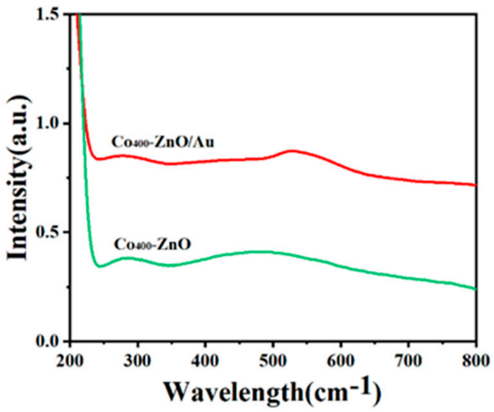

Figure S15. UV-vis diffusion reflectance spectra of Co_{400}-ZnO and Co_{400}-ZnO/Au samples.

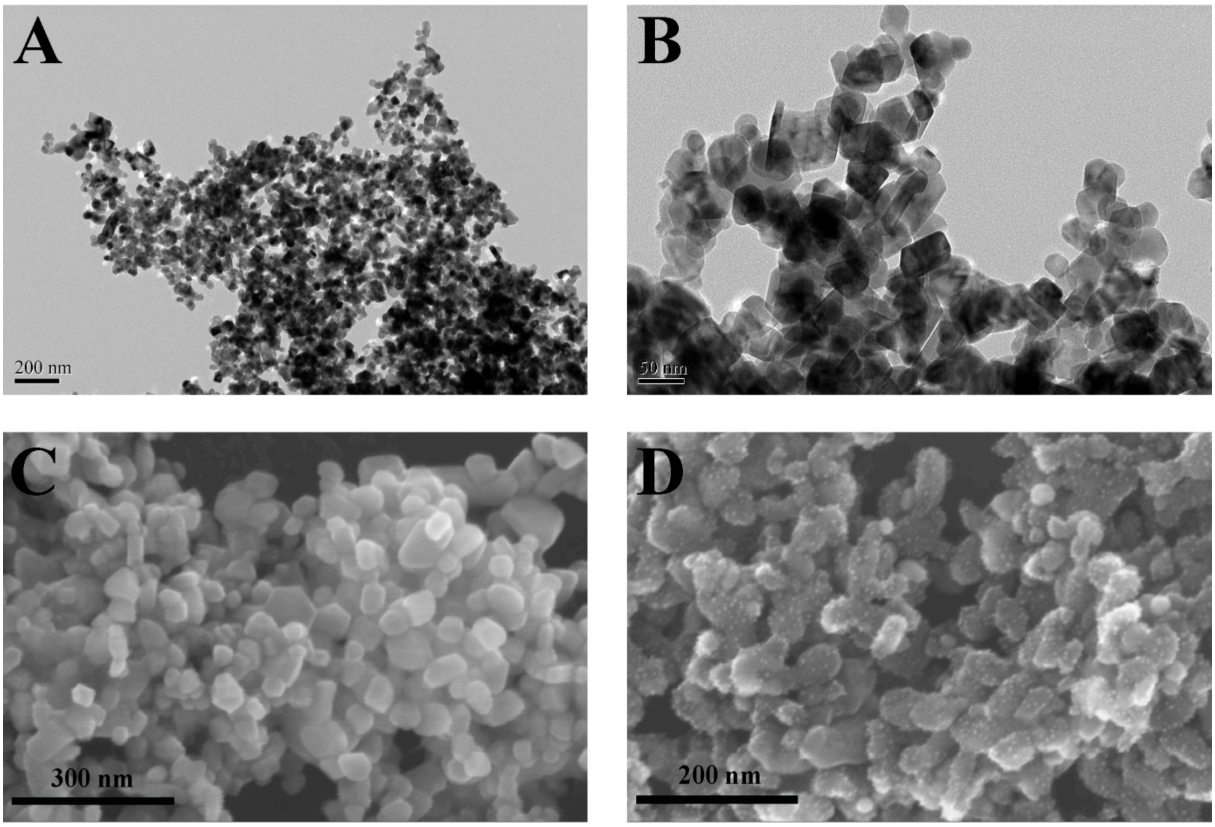

Figure S16. (A)(B) TEM images of Co_{400}-ZnO. (C) SEM image of Co_{400}-ZnO. (D) SEM image of Co-ZnO/Au.

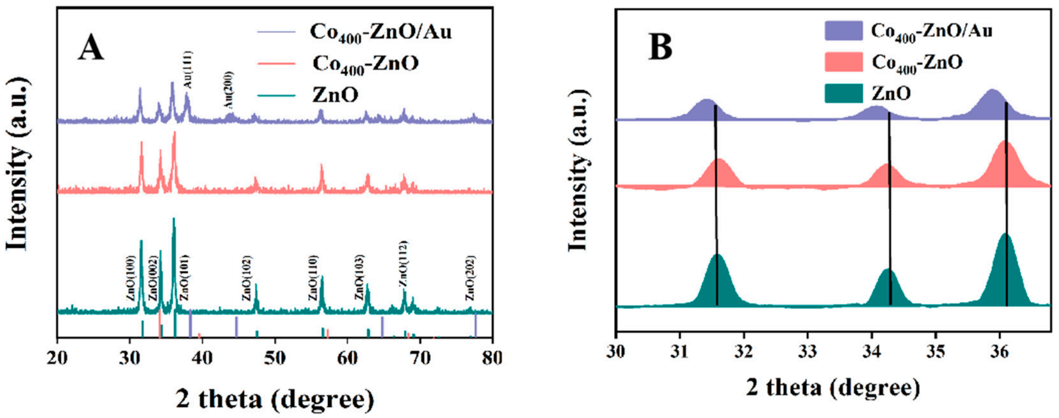

Figure S17. XRD patterns of ZnO, Co_{400}-ZnO, and Co_{400}-ZnO/Au. (A) Wide-angle patterns and (B) Selected-angle patterns.

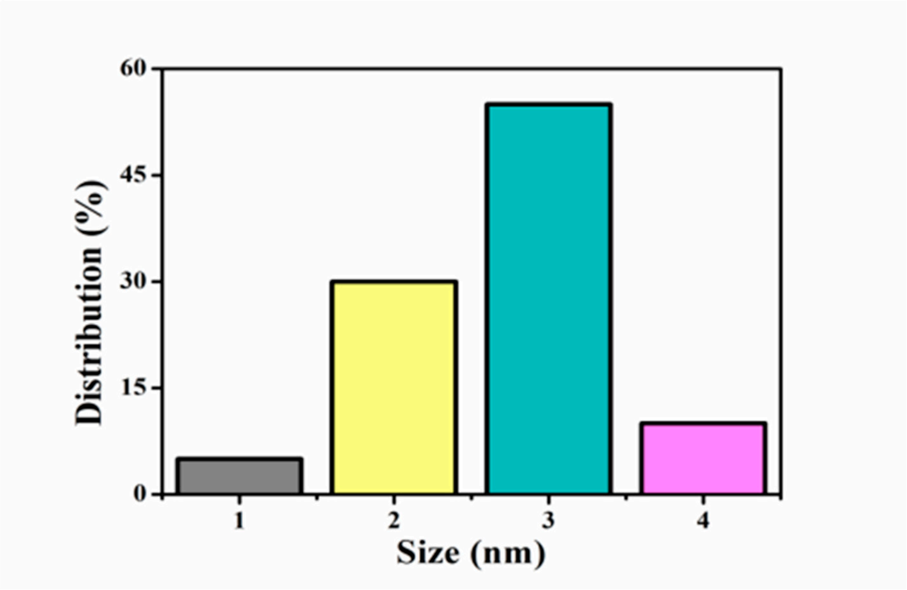

Figure S18. Size distribution of ZnO quantum dots in Co_{400}-ZnO/Au.

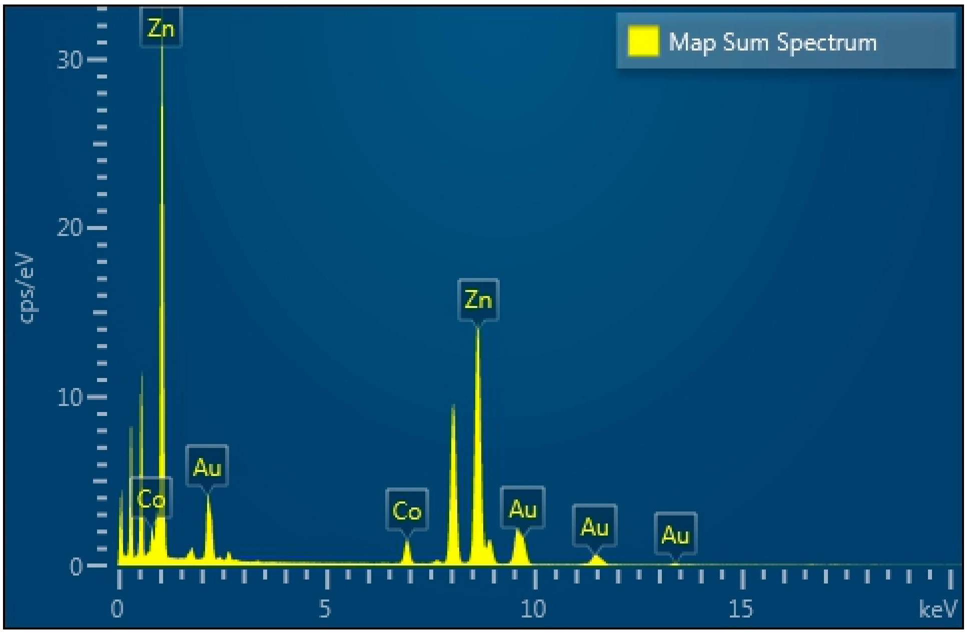

Figure S19. TEM-EDS result of Co-ZnO/Au.

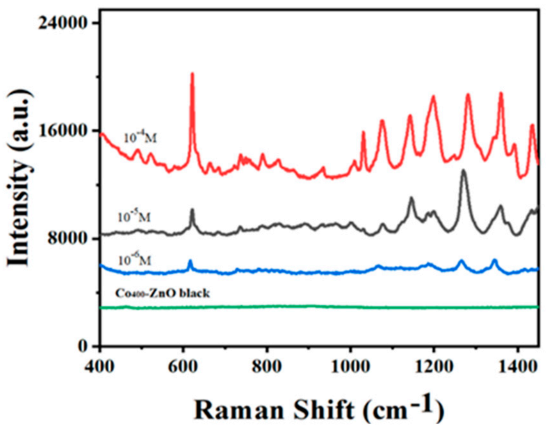

Figure S20. Concentration-dependent SERS spectra of R6G solutions recorded on Co_{400}-ZnO.

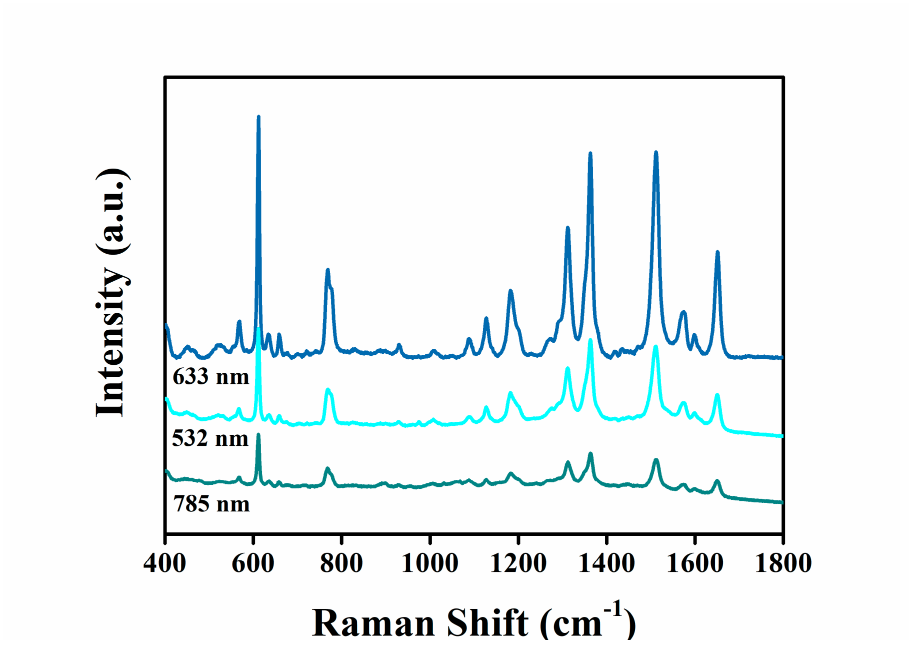

Figure S21. SERS spectra of R6G (10^{-7}) recorded on Co_{400}-ZnO/Au under different irradiations with 532, 633, and 785 nm lasers.

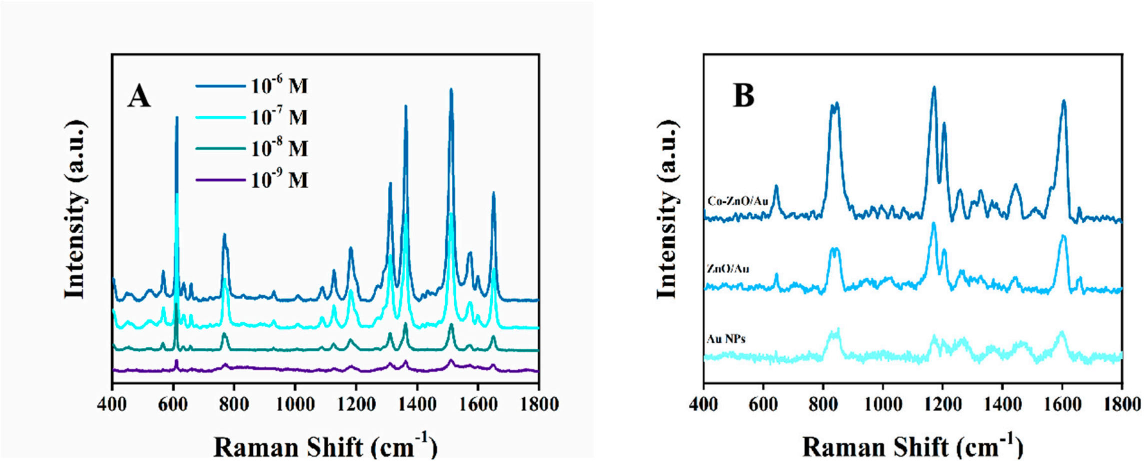

Figure S22. (A) SERS spectra of R6G molecules adsorbed onto Co-ZnO/Au. (B) SERS spectra of Tyr (1×10^{-8} M) on Co-ZnO/Au , ZnO/ Au, and Au NPs.

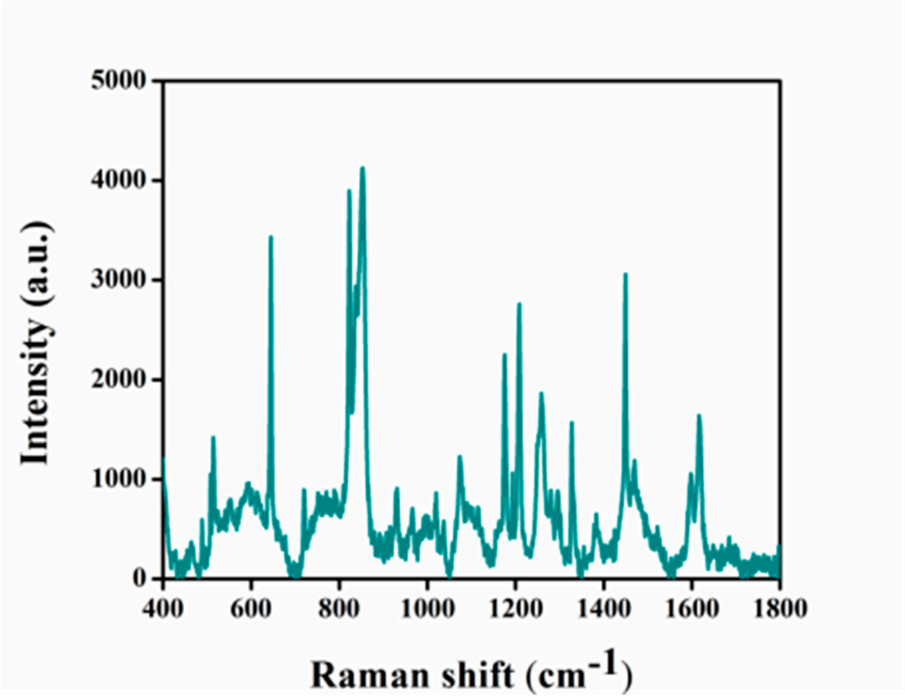

Figure S23. Normal Raman spectrum of powder Tyramine.

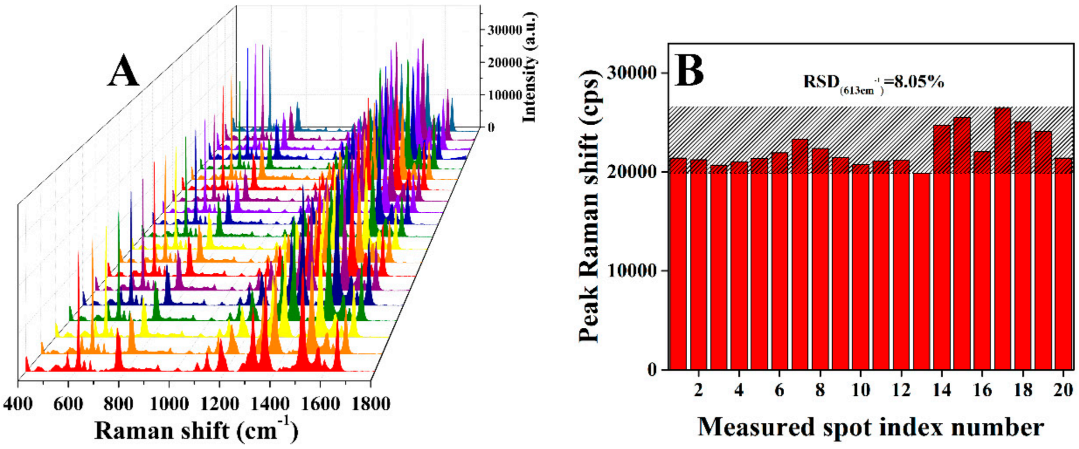

Figure S24. (A) SERS spectra of R6G (10^{-6} M) recorded from 20 randomly-selected points on Co_{400}-ZnO/Au. (B) The statistic on Raman Intensities of R6 G (10^{-6} M) recorded from 20 randomly-selected points on Co_{400}-ZnO/Au.

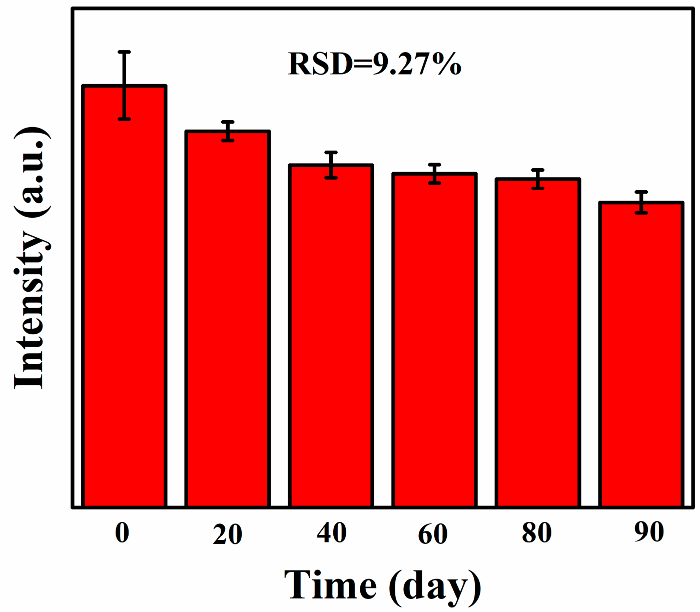

Figure S25. Raman intensities of R6G (10^{-6} M) by using Co_{400}-ZnO/Au monitored during storage in ambient condition for 90 days.

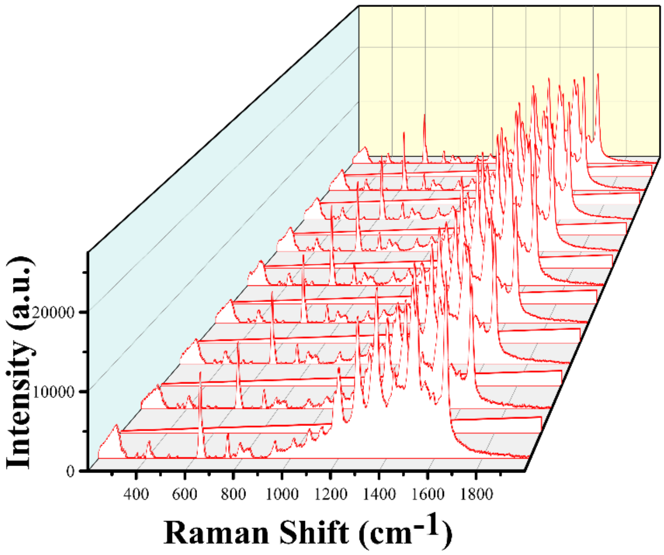

Figure S26. The SERS spectra of R6G (10^{-6} M) recorded on the three batches of Co_{400}-ZnO/Au substrate.

Table S1. Summary of Crystallite size (*D*), Dislocation density (*ρ*), and Micro strain (ε).

**sample**	**Crystallite size** **D (nm)**	**Dislocation density** $\rho\times10^{-3}$（nm^{-2}）	**Micro strain** $\varepsilon\times10^{-3}$
ZnO	27.86435	1.287960	3.997792
Co_{40}-ZnO	21.71403	2.120895	5.126717
Co_{120}-ZnO	24.51444	1.664011	4.543486
Co_{200}-ZnO	22.23124	2.023358	5.012784
Co_{280}-ZnO	24.22917	1.703424	4.598817
Co_{400}-ZnO	22.59271	1.95913	4.930610
Co_{480}-ZnO	22.96643	1.895889	4.847792
Co_{600}-ZnO	22.3514	2.001661	4.983178

Table S2. Binding energy (BE) of Co in Co-ZnO samples.

**Sample**	**Co $2p_{3/2}$ (eV)**		**Satellite (eV)**		**Co $2p_{1/2}$ (eV)**		**Co^{2+}/Co^{3+}**
	**Co^{2+}**	**Co^{3+}**	**Co^{2+}**	**Co^{3+}**	**Co^{2+}**	**Co^{3+}**	
Co_{120}-ZnO	780.6	796.38	781.12	799.56	785.53	802.95	0.865456
Co_{200}-ZnO	779.85	794.51	781.58	796.4	786.24	801.99	0.886688
Co_{280}-ZnO	779.4	794.77	780.91	796.45	786.4	802.31	0.93761
Co_{400}-ZnO	779.51	794.83	781.2	796.52	785.84	802.37	0.957815
Co_{480}-ZnO	779.55	794.52	780.81	796.65	784.78	802.26	0.753678
Co_{600}-ZnO	779.58	794.39	781.44	796.37	786.27	801.8	0.887147

Table S3. Binding energy (BE) of Zn and O in Co_x-ZnO samples.

**Sample**	**Zn 2p (eV)**		**O 1s (eV)**	
	**Zn $2p_{3/2}$**	**Zn $2p_{1/2}$**	**O_L**	**O_V**
ZnO	1021.26	1044.34	530.05	531.4
Co_{40}-ZnO	1021.20	1044.41	530.03	531.3
Co_{120}-ZnO	1021.30	1044.40	529.95	531.29
Co_{200}-ZnO	1021.20	1044.30	529.92	531.15
Co_{280}-ZnO	1021.56	1044.56	530.38	531.9
Co_{400}-ZnO	1021.44	1044.43	530	531.25
Co_{480}-ZnO	1021.52	1044.73	530.36	532.01
Co_{600}-ZnO	1021.28	1044.39	529.83	531.2

Table S4. The band gap (Eg) and optical absorption edge (nm) of pure ZnO and Co-ZnO samples.

Sample	CB (eV)	VB (eV)	Eg (eV)	optical absorption edge (nm)
ZnO	-1.06	2.18	3.24	387
Co_{40}-ZnO	-1.05	2.06	3.11	393
Co_{120}-ZnO	-0.68	2.35	3.03	395
Co_{200}-ZnO	-0.82	2.23	3.05	460
Co_{280}-ZnO	-1.41	1.7	3.11	457
Co_{400}-ZnO	-0.27	2.49	2.76	479
Co_{480}-ZnO	-0.58	2.27	2.85	465
Co_{600}-ZnO	-0.58	2.3	2.88	467

Table S5. Binding energy (BE) of ZnO, Co_{400}-ZnO and Co_{400}-ZnO/Au.

Sample	BE (eV)									
	Zn^{2+}		O		Co^{3+}		Co^{2+}		Satellite (eV)	
	$2P_{1/2}$	$2P_{3/2}$	1s	1s	$2P_{1/2}$	$2P_{3/2}$	$2P_{1/2}$	$2P_{3/2}$	$2P_{1/2}$	$2P_{3/2}$
ZnO	1044.08	1021.18	531.28	529.98	--	--	--	--	--	--
Co_{400}-ZnO	1044.43	1021.44	531.25	530.00	779.51	794.83	796.52	781.2	802.37	785.84
Co_{400}-ZnO/Au	1044.39	1021.31	531.37	530.08	779.93	795.24	796.71	781.27	802.19	785.84
